# Inferring high-fat dietary patterns from electronic health record data using machine learning

**DOI:** 10.1093/jamiaopen/ooaf181

**Published:** 2026-01-07

**Authors:** Ya-Yun Yeh, Hsin-Yueh Lin, Jingchuan Guo, Ramon C Sun, Sizun Jiang, Jiang Bian, Hao Dai

**Affiliations:** Department of Pharmaceutical Outcomes and Policy, University of Florida, College of Pharmacy, Gainesville, FL 32610, United States; Department of Pharmaceutical Outcomes and Policy, University of Florida, College of Pharmacy, Gainesville, FL 32610, United States; Department of Pharmacy Practice, Purdue University College of Pharmacy, Indianapolis, IN 46202, United States; Center for Biomedical Informatics, Regenstrief Institute, Indianapolis, IN 46202, United States; Department of Biochemistry and Molecular Biology, University of Florida, Gainesville, FL 32610, United States; Center for Advanced Spatial Biomolecule Research, University of Florida, Gainesville, FL 32610, United States; McKnight Brain Institute, University of Florida, Gainesville, FL 32610, United States; Cancer Research Institute, Beth Israel Deaconess Medical Center, Harvard Medical School, Boston, MA 02215, United States; Center for Biomedical Informatics, Regenstrief Institute, Indianapolis, IN 46202, United States; Department of Biostatistics & Health Data Science, Indiana University School of Medicine, Indianapolis, IN 46202, United States; Department of Biostatistics & Health Data Science, Indiana University School of Medicine, Indianapolis, IN 46202, United States

**Keywords:** high-fat dietary, machine learning, computable phenotyping, electronic health records

## Abstract

**Objectives:**

Electronic health records (EHRs) rarely capture dietary detail, limiting diet–disease research. We aimed to develop machine learning (ML) computable phenotypes to identify high-fat diet (HFD) using variables typically available in EHRs.

**Materials and Methods:**

We used National Health and Nutrition Examination Survey (NHANES) 1999-2020 data, where 24-h dietary recall served as ground truth. Dietary fat intake was summarized into a score (0-30) based on percent energy from fat, carbohydrate, and protein; lower scores indicated HFD. We defined HFD at cutoffs of 10, 15, and 20, and trained ML models (Extreme Gradient Boosting, logistic regression, random forest) using EHR-compatible variables (demographics, comorbidities, labs, anthropometrics). Model interpretability was assessed using Shapley Additive Explanations. To evaluate clinical relevance, we compared cancer associations using ML-predicted vs true diet labels.

**Results:**

Machine learning models classified HFD with good performance, strongest at broader definitions. Random forest achieved an F1-score of 0.79 (recall 0.74, precision 0.84) at cutoff 20. Key predictors included race/ethnicity, triglycerides, obesity metrics (body mass index and derived indices), and metabolic panel results.

**Discussion:**

These findings indicate that dietary patterns, though seldom recorded in EHRs, can be inferred from routinely available variables. The ability of ML-derived phenotypes to reproduce known diet–disease relationships underscore their epidemiologic validity. Top predictors also align with established biological pathways linking obesity, lipid metabolism, and cancer risk, supporting plausibility.

**Conclusion:**

A high-fat dietary pattern can be inferred from EHR-compatible variables using ML-based phenotyping. This approach offers a scalable tool to integrate diet into EHR-based research and precision medicine.

## Introduction

Dietary behavior is a fundamental contributor to chronic disease risk,^1^^,^^2^ yet detailed diet information is rarely recorded in structured fields of electronic health records (EHRs).^3^ Instead, dietary factors are inferred indirectly through markers like obesity diagnoses, laboratory abnormalities, or documentation of lifestyle counseling, rather than being explicitly recorded.^4^ This limitation constrains our ability to study diet–disease relationships at scale and hinders the integration of dietary behavior into precision medicine frameworks.^5^

To address these challenges, medical informatics research has turned to the development of “computable phenotypes,”^6^ proxy indicators derived from structured and unstructured EHR data to approximate lifestyle factors like diet. Prior studies have leveraged natural language processing (NLP) and machine learning (ML) to identify documentation of diet and exercise counseling from clinician notes,^7^ or to track nutrition-related care events such as preventive visits and distribution of dietary educational materials.^7^ In metabolic disease research, diet is often acknowledged only implicitly, with models relying on surrogates such as lipid profiles, body mass index (BMI), or diagnoses like hyperlipidemia to approximate dietary exposure.^8^

However, no existing approach has directly classified patients’ dietary patterns, such as high-fat intake at scale using EHR-based features, due in part to the lack of ground-truth dietary data in routine clinical care. Existing proxies have not been systematically validated against detailed dietary intake, and most prior efforts have focused on narrow populations or outcomes, limiting generalizability. Moreover, methodological rigor has varied, with some models relying on rule-based heuristics or limited validation cohorts. Approaches that depend heavily on clinical notes also face substantial limitations, as performance is highly sensitive to note quality, documentation practices, and the extraction pipeline used, which often requires site-specific customization and may hinder scalability.^9^

In this study, we propose a data-driven approach to address these gaps. Leveraging the National Health and Nutrition Examination Survey (NHANES), which provides comprehensive 24-h dietary recall data, we trained ML-based models to identify individuals with a high-fat diet using only features commonly available in EHRs. By aligning structured clinical data with real dietary intake, we constructed a computable phenotype for high-fat diet exposure and evaluated its predictive performance. This model captures complex, nonlinear relationships between metabolic markers and diet, offering a robust framework for inferring dietary patterns in settings where direct dietary information is unavailable.

Our work extends the capabilities of EHR-based phenotyping by introducing a validated, computable proxy for high-fat diet, a lifestyle risk factor not typically encoded in medical records. This approach may enable more comprehensive risk stratification in clinical and research settings, facilitating the integration of diet into population health studies, predictive modeling, and targeted interventions.

## Methods

### Data source and study population

We utilized data from the NHANES from 1999 to 2020 to construct the study cohort.^10^ NHANES is a nationally representative survey designed to assess the health and nutritional status of the US population, incorporating health examinations, laboratory tests, and dietary interviews for participants of all ages. It employs a complex, stratified, multistage probability sampling design, which includes the oversampling of specific subgroups (eg, racial/ethnic minorities and older adults) to ensure reliable estimates. To address potential selection bias, nonresponse, and the unequal probability of selection, NHANES provides specific survey weights that allow for the generation of nationally representative estimates. For this study, we included participants aged 20 years or older who completed a mobile examination center (MEC) assessment and had valid day 1 dietary data. As with all survey-based dietary data, results may be influenced by nonresponse and measurement error.

### Dietary assessment

National Health and Nutrition Examination Survey dietary interview was conducted in person at the MEC, where participants reported all foods and beverages consumed in the preceding 24 h.^11^ Beginning in 2003, a second 24-h dietary recall was administered by telephone approximately 3-10 days after the initial interview. For this study, we used day 1 total energy intake for the 2003-2020 period and total energy intake for 1999-2002 to calculate the dietary fat intake score, as day 2 dietary data were not available for the earlier years.

### Outcomes: dietary fat intake score

Dietary patterns were evaluated using a dietary fat intake score, derived from NHANES dietary interview data and adapted from a previously published “low-fat diet score” methodology.^12^ Participants’ energy intake from each macronutrient (carbohydrate, protein, and fat) was first determined. Subsequently, individuals were categorized into 11 specific strata for each macronutrient, based on the percentage of total energy derived. For carbohydrate and protein, participants in the highest stratum received 10 points, while those in the lowest received 0 points. Conversely, for fat intake, the scoring was inverted, with the lowest stratum receiving 10 points and the highest receiving 0 points. The 3 components were summed to yield a total score:


$${\text{DFIS}}_{i}=S_{i}^{\left(\mathrm{carb}\right)}+S_{i}^{\left(\mathrm{prot}\right)}+S_{i}^{\left(\mathrm{fat}\right)}$$

yielding a total range of 0-30, where lower scores indicate higher dietary fat intake.

For analytic purposes, we dichotomized the score at 3 thresholds (10, 15, and 20). Scores below each cutoff were classified as high-fat diet (HFD = 1) and scores at or above the cutoff as low-fat diet (HFD = 0). These thresholds were prespecified to capture increasing stringency of the HFD definition and to facilitate sensitivity analyses across multiple epidemiologically interpretable cut points. Prior studies using the same 0-30 diet score have operationalized exposure using quantiles (eg, quartiles) and linked higher adherence to clinical outcomes (including cancer risk), providing an established benchmark for interpreting different levels of the score.^13^

### Cancer variables

To evaluate epidemiologic validity, we examined prevalent cancer outcomes derived from the NHANES Medical Conditions Questionnaire. Participants were first asked whether they had ever been told by a doctor or other health professional that they had “cancer or a malignancy of any kind” (variable MCQ220). Those who responded “yes” were then asked, for up to 4 primary cancers, “What kind of cancer was it?” (variables MCQ230A-D).

We defined any cancer as a “yes” response to MCQ220. Site-specific outcomes (colorectal, breast, lung, prostate, and a composite urologic cancer endpoint) were constructed from the reported cancer sites in MCQ230A-D. All cancer outcomes therefore represent self-reported, physician-diagnosed malignancies captured within NHANES; no external EHR or registry linkage was used.

### Predictor candidates

We assembled a comprehensive set of variables from NHANES. Demographic information (age, sex, race/ethnicity) was obtained through household interviews, and dietary intake variables (eg, total energy, carbohydrate, protein, and fat intake) were derived from 24-h dietary recalls. Examination data provided BMI, systolic and diastolic blood pressure, while other laboratory test values (25 clinical chemistry panel measures; details in Table S1) were collected from the MEC examination. Medical conditions, including diabetes, prediabetes, asthma, overweight, arthritis, heart failure, coronary heart disease, angina, myocardial infarction, stroke, liver disease, hypertension, high cholesterol, kidney disease, chronic obstructive pulmonary disease, and cancers (breast, lung, uterus), were defined based on self-reported physician diagnoses. Prescription medication use over the past 30 days was self-reported and mapped to therapeutic categories(36 observed in our data) using the Multum Lexicon system (eg, analgesics, antidepressants, antidiabetic agents, cardiovascular medications). In addition, one lifestyle factor (smoking) was included.

### Development of ML pipeline for high-fat diet prediction models

**Figure 1.**
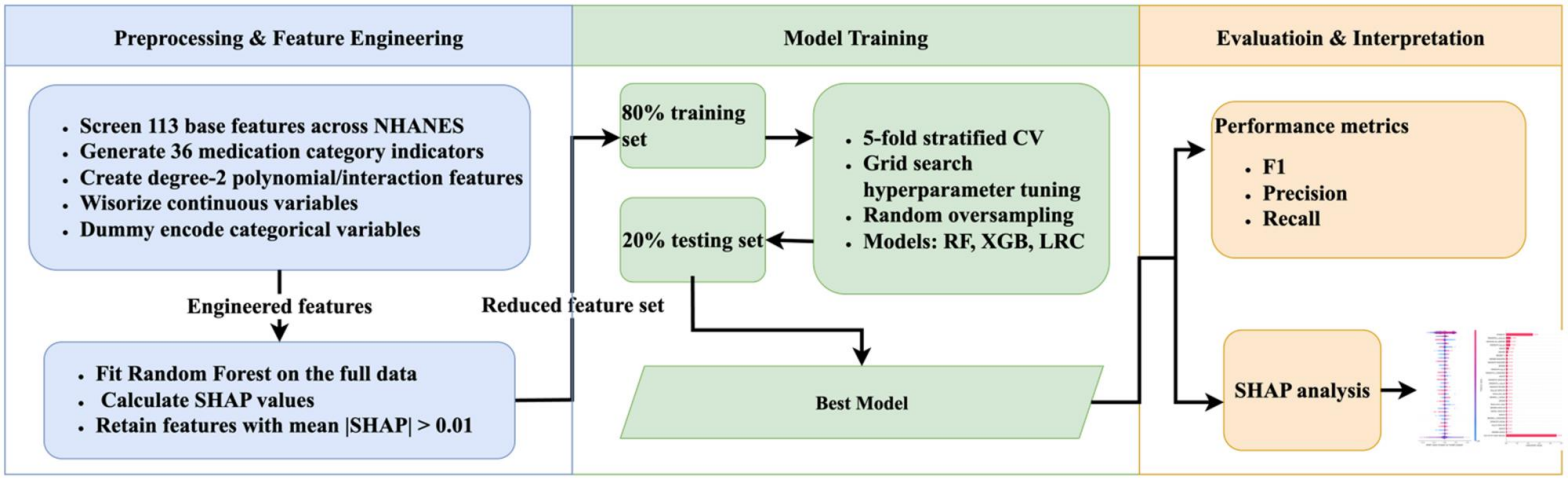
The workflow for ML pipeline. Abbreviation: ML, machine learning.

The workflow for ML pipeline is shown in Figure 1, including 4 main steps: data preprocessing and feature engineering, model training, performance evaluation, model interpretation.

Given the clinical objective of this study is to derive an actionable, interpretable model linking routinely collected physiological biomarkers to dietary patterns. We prioritized modeling approaches that perform well on structured clinical data while supporting transparent interpretation. Several widely used ML models were employed to predict the HFD status, categorized into linear and tree-based models. For the linear models, we adjusted a range of hyperparameters and penalty functions for logistic regression classifier (LRC). For the tree-based models, we selected random forest (RF) and Extreme Gradient Boosting (XGB) as our primary modeling techniques based on their proven efficacy with structured, tabular data. Recent benchmarks demonstrate that tree-based ensemble methods (XGB, RF) consistently achieve strong performance on tabular datasets,^14–17^ often outperforming deep neural networks while requiring less computational resource for hyperparameter tuning.^18^^,^^19^ In addition, these models integrate naturally with Shapley Additive Explanations (SHAP)-based explanations, enabling consistent quantification of feature contributions and facilitating clinically meaningful interpretation of the predictors driving dietary phenotype classification.

#### Data preprocessing and feature engineering

We screened predictors for availability across NHANES cycles, this yielded 113 available base features. We then derived medication-category indicators (36 observed in the data) and degree-2 polynomial/interaction features, resulting in 704 engineered features. The degree-2 terms were included to capture clinically plausible nonlinearities and synergistic effects among biomarkers that are known to relate to dietary fat intake and metabolic status (eg, lipid profiles, adiposity-related measures, glucose–insulin dysregulation, and inflammatory markers), which may not be well represented by main effects alone.^20–22^ Continuous predictors were winsorize to reduce the influence of extreme values. We conduct imputation by mean value to allow algorithms that do not accept missingness (logistic regression) to be fit on complete-case data. Selected categorical variables (eg, sex, race/ethnicity, smoking) were dummy encoded via pandas; missing categories were not explicitly encoded as a separate level.

For post hoc interpretation and feature screening, we first fit an RF on the full analytic sample for each cutoff and compute SHAP^23^ values. Features with mean absolute SHAP>0.01 were retrained. Using this reduced feature set, we then performed a new random 80/20 train-test spilt, trained the models on the training spilt, and evaluated them on the heldout test spilt.

#### Model training

We implemented RF, XGB, and LRC. For each cutoff, hyperparameters were tuned within the 80% training split via stratified 5-fold cross-validated grid search (*RF: n_estimators, max_depth, max_features; XGB: n_estimators, max_depth, learning_rate, reg_alpha, reg_lambda; LRC: C, penalty, solver*). To address class imbalance during tuning, we applied random oversampling inside each training fold; for XGB, we also considered *scale_pos_weight*. F1-score was the primary selection metric (with early stopping for XGB). The selected model was refitted on the full training split and evaluated on the heldout 20% test set.

In addition to the random train–test split, we performed a temporal validation by training models on NHANES 1999-2016 (SDDSRVYR 1-9) and evaluating them on the independent 2017-2020 prepandemic cycle. We report F1-score, recall, and precision for the heldout test set.

#### Performance evaluation

Model performance was assessed using a set of complementary metrics. F1-score was calculated as the harmonic mean of precision and recall, providing a balanced measure of classification performance. Precision measured the proportion of positive predictions that were correct, while recall measured the proportion of actual positives that were correctly identified.

#### Model interpretation

Shapley Additive Explanations were applied to interpret the ML models. Feature importance was quantified using SHAP values, which represent the marginal contribution of each feature to the models’ predictions. The top features were ranked by their mean absolute SHAP values, and importance scores were aggregated by feature categories (eg, medication, laboratory, demographic, and health conditions) to understand relative contributions. Feature category analysis involved calculating aggregate importance scores across medication features, health conditions, laboratory values, and demographic variables.

### Statistical analysis about association of high-fat diet with cancer outcomes

To evaluate the clinical validity of the predicted dietary patterns, we examined their associations with cancer outcomes and compared them with the associations observed for the true dietary patterns. For each cancer outcome, logistic regression models were fitted, adjusting for relevant demographic and clinical covariates, specifically age, sex, race/ethnicity, and smoking status, which are established confounders in cancer epidemiology.^24^^,^^25^

First, we established the ground-truth association by fitting a logistic regression model using the true HFD label as the exposure. The resulting regression coefficient was exponentiated to obtain the odds ratio (OR) for the association between the true HFD label and cancer, along with the corresponding 95% CI and *P*-value.

Second, we evaluated the predicted label association by fitting a similar logistic regression model using the predicted HFD label as the exposure. The OR, 95% CI, and *P*-value were derived in the same manner as for the ground truth model.

Finally, we compared the direction and magnitude of these associations. Directional consistency was defined as both the true and predicted associations being either positive (OR>1) or negative (OR<1). Magnitude consistency was assessed by calculating the ratio of the predicted OR to the true OR. Ratios close to one, combined with overlapping 95% CIs, were interpreted as strong agreement between the predicted and true associations.

Machine learning models were implemented using the scikit-learn library for logistic regression and RF, and the XGB library for gradient boosting in Python. Statistical analyses were conducted using the statsmodels package.

## Results

The final analytic cohort consisted of 51 698 questionnaire respondents (Table S1). The mean age was 49.6 ± 18.1 years, and 51.8% were female. The racial/ethnic breakdown was 44.3% non-Hispanic White, 17.2% non-Hispanic Black, 21.5% Hispanic, and 8.3% other. Non-Hispanic Black and Hispanic participants were younger than non-Hispanic Whites (46.1 and 48.6 years vs 52.6 years, respectively).

In descriptive analyses (Table S2), we further compared participants classified as high-fat vs nonhigh-fat diet across demographic characteristics. Participants classified as having a high-fat diet were broadly similar to those in the nonhigh-fat diet group with respect to age, BMI, and most cardiovascular comorbidities. However, the distribution of high-fat diet classification varied across race/ethnicity, with a higher proportion of non-Hispanic White and non-Hispanic Black participants in the high-fat diet group.

### Predictive model performance

We evaluated 3 supervised ML algorithms, XGB, LRC, and RF, using Radom oversampling at 3 label-count cutoffs (10, 15, and 20). Table  1 presents the results after SHAP-based feature selection. At cutoff 10, XGB and LRC reached F1-scores of 0.415 and 0.419, respectively, while RF scored 0.396. At cutoff 15, XGB achieved 0.601, LRC 0.596, and RF 0.618. At cutoff 20, RF again delivered the best performance (0.787), followed by XGB (0.714) and LRC (0.714), while maintaining strong recall (0.739) and precision (0.84).

**Table 1.** Performance metrics for XGB, LRC, and RF on testing set.

Cutoff	Model	F1	Recall	Precision
10	XGB	0.415 ± 0.009	0.606 ± 0.044	0.317 ± 0.011
	LRC	0.419 ± 0.006	0.587 ± 0.011	0.326 ± 0.005
	RF	0.396 ± 0.008	0.470 ± 0.016	0.342 ± 0.007
15	XGB	0.601 ± 0.014	0.615 ± 0.035	0.589 ± 0.010
	LRC	0.596 ± 0.005	0.593 ± 0.007	0.599 ± 0.004
	RF	0.618 ± 0.005	0.643 ± 0.010	0.595 ± 0.005
20	XGB	0.714 ± 0.012	0.618 ± 0.019	0.847 ± 0.005
	LRC	0.714 ± 0.005	0.615 ± 0.006	0.851 ± 0.003
	RF	0.787 ± 0.005	0.739 ± 0.009	0.840 ± 0.003

Abbreviations: LRC, logistic regression classifier; RF, random forest; XGB, Extreme Gradient Boosting.

In temporal validation, performance was modestly lower than in random split validation, as expected, but remained within an acceptable range (Table S3). For example, at an HFD cutoff of 20, the RF model achieved an F1-score of 0.73 in the temporal test set, compared with 0.78 in the random split test.

### Important predictors of high-fat diet

**Figure 2.**
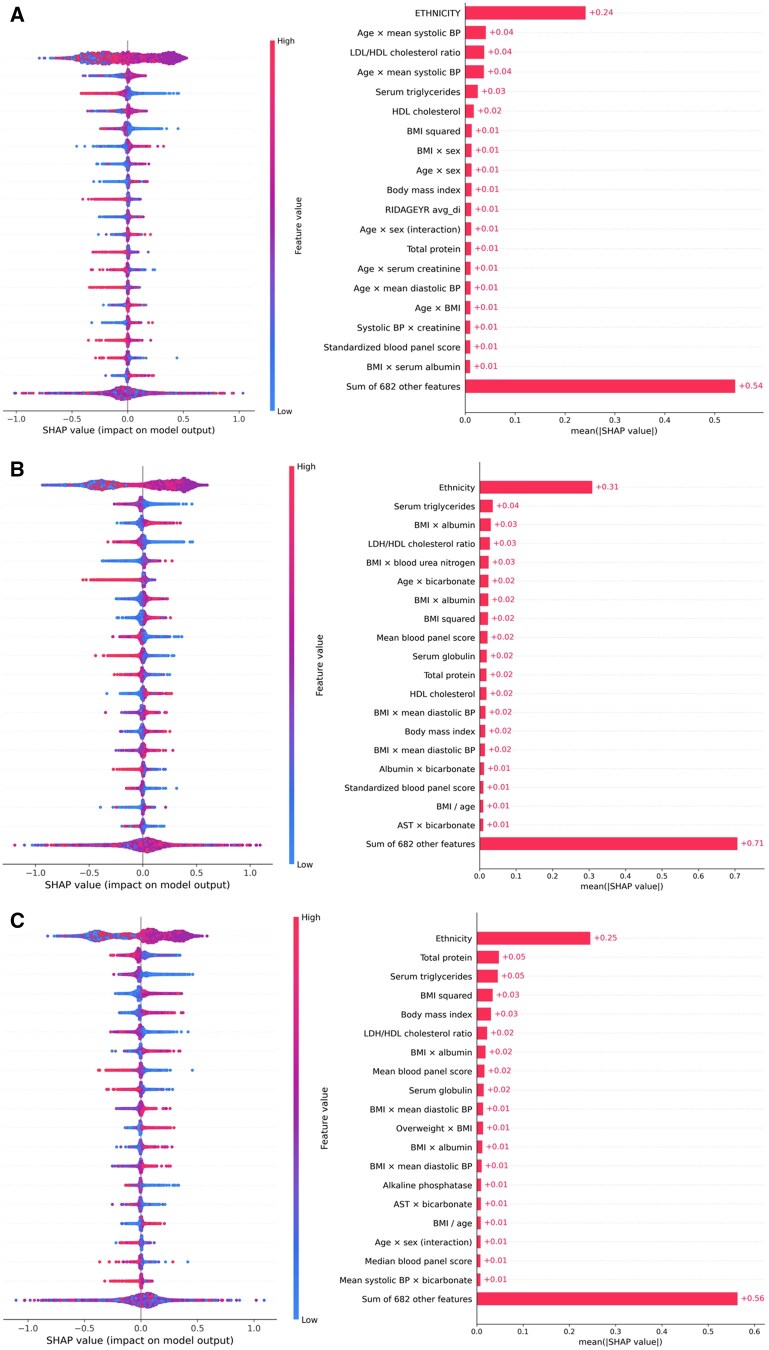
SHAP values of important predictors based on RF model: (A) cutoff 10, (B) cutoff 15, and (C) cutoff 20. Abbreviations: RF, random forest; SHAP, Shapley Additive Explanations.

Our SHAP values showed that the top-ranked laboratory biomarkers were total protein, serum triglycerides, and BMI (see Figure 2). Although the contribution of other individual features beyond the top-ranked variables was smaller, their cumulative effect was substantial, as reflected by the summed SHAP value for the remaining features.

### Associations with cancer outcomes

Using a cutoff of 20 for the dietary fat intake score, we evaluated the associations of actual and predicted HFD status with multiple cancer outcomes (Table  2), ORs, CIs, *P*-values, effect size ratios, and association directions are summarized. Across most cancer endpoints, the ORs for predicted labels were directionally consistent with those for the actual labels.

**Table 2.** Association between true and predicted high-fat diet labels and cancer outcomes.

Outcome	True OR (95% CI)	*P*-value	Pred OR (95% CI)	*P*-value	Effect ratio	Direction
Cancer	1.23 (1.03-1.47)	.02	1.57 (1.33-1.84)	.00	1.27	Same
Colorectal cancer	1.17 (0.68-2.00)	.57	1.15 (0.71-1.85)	.57	0.99	Same
Urologic cancer	1.18 (0.81-1.73)	.39	1.68 (1.00-2.41)	.01	1.42	Same
Breast cancer	0.86 (0.59-1.27)	.46	0.87 (0.61-1.24)	.43	1.00	Same
Lung cancer	1.21 (0.41-3.60)	.73	1.01 (0.39-2.62)	.99	0.83	Same
Prostate cancer	1.13 (0.74-1.73)	.58	1.50 (1.00-2.25)	.05	1.33	Same

*P*-values, effect size ratios, and association directions are shown for both true and predicted labels.

Abbreviation: OR, odds ratio.

For colorectal cancer, the true label OR was 1.17 (95% CI, 0.68-1.99, *P* = .57), while the predicted label OR from the RF model was 1.15 (95% CI, 0.71-1.85, *P* = .57), yielding an effect ratio of 0.98 with the same positive direction. Urologic cancers had a true label OR of 1.18 (95% CI, 0.81-1.73, *P* = .38) and a predicted OR of 1.68 (95% CI, 1.17-2.41, *P* = .005), with an effect ratio of 1.42, again directionally consistent. Breast cancer showed a true OR of 0.86 (95% CI, 0.59-1.27, *P* = .46) and a predicted OR of 0.87 (95% CI, 0.61-1.24, *P* = .43), with an effect ratio of 1.00, indicating consistent negative association direction.

For lung cancer, the true OR was 1.21 (95% CI, 0.41-3.60, *P* = .73), and the predicted OR was 1.01 (95% CI, 0.39-2.62, *P* = .99), with an effect ratio of 0.83, both positive in direction. Prostate cancer demonstrated a true OR of 1.13 (95% CI, 0.74-1.73, *P* = .57) and a predicted OR of 1.50 (95% CI, 1.00-2.25, *P* = .049), with an effect ratio of 1.33, all in the same positive direction. Endometrial cancer showed a true OR of 2.05 (95% CI, 1.12-3.76, *P* = .02) and a predicted OR of 1.44 (95% CI, 0.88-2.36, *P* = .15), with an effect ratio of 0.70, directionally consistent. For overall cancer, the true OR was 1.23 (95% CI, 1.03-1.47, *P* = .02), and the predicted OR was 1.57 (95% CI, 1.33-1.84, *P* < .0001), with an effect ratio of 1.27, maintaining the same positive association direction.

A post hoc power analysis indicated that the study had limited statistical power to detect modest associations for individual cancer subtypes (eg, lung cancer) given the observed effect sizes and sample prevalence (see details in Table S4).

## Discussion

In this study, we developed and validated an ML-based approach to infer individuals’ dietary fat intake patterns from variables readily available in EHRs. Our results demonstrate that despite the absence of explicit dietary data in clinical records, an ML model can leverage demographic, anthropometric, and laboratory information to approximate a patient’s diet quality. The model’s performance improved as more lenient definitions of “high-fat diet” were used, achieving nearly 79% F1-score in identifying patients with relatively high fat consumption. Overall, the ML-predicted dietary labels exhibited epidemiologic concordance with dietary recall-derived labels: across cancer outcomes, the associations estimated using ML-predicted labels closely matched those based on measured dietary labels in direction and magnitude. Notably, high-fat diet was associated with higher risk for overall cancer and several site-specific cancers, whereas breast cancer showed a modest inverse (nonsignificant) association under both labeling approaches. The associations pointed in the same directions for individual cancer types, though not statistically significant. Based on the post hoc power analysis, the lack of statistical significance for certain low-prevalence cancers likely reflects limited statistical power in NHANES. This finding indicates that the computable diet phenotype captures meaningful variation in risk-related behavior, rather than being an arbitrary artifact of the data or modeling process. The main contribution here is a scalable phenotype definition that can be applied to larger EHR datasets where adequate power is available for these outcomes.

Our work builds on and extends prior efforts in EHR-based lifestyle phenotyping. Earlier studies have identified documentation of diet or exercise in clinical text and tracked nutritional counseling events,^4^^,^^7^^,^^26^ but these approaches capture only whether diet was mentioned, not an underlying dietary pattern. Other research has used biomarkers or diagnoses as crude surrogates for diet.^8^ In contrast, our approach directly classifies a high-fat dietary intake pattern by training on objective dietary intake data and translating those patterns to the EHR domain. By aligning NHANES with the types of data found in EHRs, we created a proxy that is both data-driven and clinically interpretable. To our knowledge, this is the first validated model to infer an overall diet quality metric (high-fat vs low-fat diet) from routine health records at scale. This represents a step toward integrating lifestyle factors into clinical big data research, enabling investigation of diet–disease relationships in populations where traditional dietary assessment is not feasible.

There are several clinical and public health implications of being able to infer dietary habits from EHR data. Clinically, an automated diet phenotype could flag patients with unhealthy eating patterns (eg, high-fat diet) for targeted nutrition interventions,^27^ without requiring additional screening questionnaires.^28^ For instance, integrating a high-fat diet indicator into EHR systems could help clinicians identify high-risk individuals during primary care visits and initiate diet counseling or refer them to dietitians proactively.^29^ It could also inform precision medicine efforts by adding a missing lifestyle dimension to patient risk profiles, including complementing genetic, metabolic, and environmental information with diet.^30^ From a population health perspective, the capability to estimate diet quality across thousands of patients opens new opportunities for monitoring and prevention.^31^ Healthcare organizations and public health agencies could use EHR-based diet phenotypes to study diet-related disease trends,^32^^,^^33^ evaluate the impact of dietary interventions at scale, or incorporate dietary risk stratification into screening programs, for example, identifying patients at elevated risk of certain cancers who might benefit from more intensive lifestyle modification support.^34^

In epidemiologic research, the availability of a diet proxy from EHR data can greatly expand study possibilities. Large retrospective cohorts that lack dietary survey data could still examine nutrition-related hypotheses by using the ML-derived diet scores. For example, one could study relationships between diet and long-term outcomes like cancer or cardiovascular disease in EHR-linked biobanks or administrative datasets by applying our model to predict diet for those patients. This approach provides a *scalable and cost-effective* alternative to conducting de novo dietary assessments. It also facilitates studying diet in subpopulations typically underrepresented in nutrition research (since EHR data often include diverse and medically vulnerable groups not well covered by surveys). Ultimately, coupling ML-derived diet phenotypes with disease registries and surveillance data (as we demonstrated for cancer outcomes) offers a sustainable strategy for monitoring diet–disease associations at the population level over time.

Several limitations should be considered. First, our dietary fat intake labels were derived from a single 24-h dietary recall (day 1). While this approach was chosen to ensure methodological consistency across the full study period (1999-2020), as second-day recalls were not available in NHANES cycles prior to 2003, we acknowledge that a single snapshot may not fully capture long-term habitual diet due to intraindividual variability. However, in the context of ML, such variability functions as “label noise.” The fact that our models achieved robust predictive performance despite this noise suggests that the physiological signal linking EHR features to dietary patterns is strong. Second, regarding the clinical predictors, we acknowledge that physiological variables such as BMI, laboratory values, and comorbidities are dynamic and evolve over time. Ideally, longitudinal trajectories would provide a more comprehensive view of health status. However, the NHANES design is inherently cross-sectional, precluding the use of historical trajectory data for the majority of participants. To ensure scientific rigor within these constraints, we prioritized temporal synchronization by utilizing physical and laboratory measurements obtained concurrently with the dietary recall interview. Consequently, our variables represent a validated “snapshot” of the participant’s metabolic state at the exact time their dietary pattern was recorded. While this approach limits the ability to model causal progression or long-term fluctuations, it remains methodologically sound for the specific objective of this study: inferring the association between a patient’s current physiological phenotype and their current dietary intake. Additionally, as the predictive models were trained using a single survey dataset (NHANES), generalizability to other populations, healthcare systems, and EHR infrastructures remains uncertain. External validation is therefore needed, particularly in EHR settings where differences in laboratory protocols (assays, units, reference ranges), coding practices (eg, ICD mapping and documentation), and missingness/measurement frequency may introduce dataset shift and degrade discrimination and calibration. Prior to deployment, models should be externally validated and assessed for calibration (with recalibration if needed), with periodic monitoring for drift over time. Furthermore, our study focused primarily on clinical motivation rather than detailed technical implementation or deployment planning. However, we provided practical deployment considerations in Supplementary Method S1, including recommendations for external validation in independent EHR systems, calibration and subgroup performance checks, and monitoring for dataset shift over time. These steps are necessary before the phenotype can be used for routine clinical decision support. Finally, while SHAP values improve interpretability, they do not establish causal relationships and should be interpreted as correlational measures of feature influence.

## Conclusion

We developed and validated ML-based computable phenotypes for high-fat diet exposure using features commonly available in EHRs, trained on NHANES data with detailed dietary recalls. The resulting ML-predicted dietary labels closely reproduced the cancer association patterns observed with actual dietary intake for most cancer outcomes, demonstrating both epidemiologic validity and clinical relevance. By leveraging structured clinical variables and advanced feature selection, our approach captures complex, multidimensional signals of dietary patterns without requiring direct dietary assessment.

These findings highlight the potential of ML-derived diet phenotypes to bridge the gap between population-based nutrition research and large-scale EHR datasets, enabling dietary risk stratification in settings where traditional dietary data are unavailable. Such phenotypes can support cancer epidemiology, inform preventive care strategies, and facilitate precision health initiatives at scale.

## Supplementary Material

ooaf181_Supplementary_Data

## Data Availability

The data used in this study are publicly available from the National Health and Nutrition Examination Survey program, conducted by the US Centers for Disease Control and Prevention. The datasets can be accessed and downloaded at: https://www.cdc.gov/nchs/nhanes. The code publicly available on GitHub (https://github.com/Throwfox/High-fat-diet-pattern-computational-phenotype)
