# Supplementary material for: Inferring high-fat dietary patterns from electronic health record data using machine learning
Source: JAMIA Open. 2026 Jan 7;9(1):ooaf181. doi: 10.1093/jamiaopen/ooaf181 (PMC12794014; doi:10.1093/jamiaopen/ooaf181)
Supplement: ooaf181_Supplementary_Data [file ooaf181_supplementary_data.docx]

Supplement

Table S1a. **Baseline characteristics—continuous variables:** mean (SD), median [Q1–Q3].

| **Variable codes** | **Mean** | **SD** | **Median** | **Q1** | **Q3** | **Min** | **Max** |
| --- | --- | --- | --- | --- | --- | --- | --- |
| BMI (BMXBMI) | 29.1 | 6.9 | 28 | 24.3 | 32.5 | 17.9 | 130.2 |
| BMI × SEX (BMXBMI RIAGENDR) | 44.4 | 19.1 | 41.0 | 28 | 57 | 17.9 | 184.6 |
| BMI^2^ (BMXBMI^2) | 891.6 | 468.2 | 784 | 595.4 | 1047.2 | 321.1 | 16954.6 |
| BMI / age (BMXBMI_div_RIDAGEYR) | 0.68 | 0.32 | 0.6 | 0.44 | 0.85 | 0.21 | 3.7 |
| BMI × sex (BMXBMI_x_RIAGENDR) | 44.4 | 19.2 | 41 | 28 | 57.2 | 17.9 | 184.6 |
| HDL cholesterol (LBDHDD) | 52.9 | 16.1 | 50 | 41 | 62 | 26 | 226 |
| LDL cholesterol (LBDLDL) | 115.2 | 35.6 | 112 | 90 | 137 | 52 | 629 |
| Serum albumin (LBXSAL) | 4.2 | 0.36 | 4.2 | 4 | 4.5 | 3.1 | 5.7 |
| Alkaline phosphatase (LBXSAPSI) | 72.3 | 27.1 | 68 | 56 | 84 | 33 | 1378 |
| Aspartate aminotransferase (LBXSASSI) | 25.1 | 18.4 | 22 | 19 | 27 | 12 | 1672 |
| Alanine aminotransferase (LBXSATSI) | 25.0 | 24.1 | 20 | 16 | 28 | 8 | 1997 |
| Blood urea nitrogen (LBXSBU) | 13.8 | 6.1 | 13 | 10 | 16 | 5 | 122 |
| Serum bicarbonate (LBXSC3SI) | 24.8 | 2.4 | 25 | 23 | 26 | 19 | 43 |
| Serum calcium (LBXSCA) | 9.4 | 0.38 | 9.4 | 9.2 | 9.6 | 8.5 | 14.8 |
| Total cholesterol (LBXSCH) | 195.4 | 42.5 | 192 | 166 | 221 | 112 | 712 |
| Serum chloride (LBXSCLSI) | 103.24 | 2.96 | 103 | 101 | 105 | 95 | 120 |
| Serum creatinine (LBXSCR) | 0.9 | 0.47 | 0.83 | 0.7 | 1 | 0.4 | 17.8 |
| Serum globulin (LBXSGB) | 2.99 | 0.46 | 2.9 | 2.7 | 3.3 | 2 | 7.9 |
| Serum glucose (LBXSGL) | 101.5 | 38 | 92 | 85 | 103 | 65 | 777 |
| Gamma-glutamyl transferase (LBXSGTSI) | 30.1 | 45.2 | 20 | 14 | 31 | 7 | 2274 |
| GGT / alkaline phosphatase ratio (LBXSGTSI_div_LBXSAPSI) | 0.42 | 0.46 | 0.31 | 0.21 | 0.47 | 0.01 | 19.7 |
| Serum iron (LBXSIR) | 85.14 | 35.6 | 81 | 61 | 105 | 20 | 476 |
| Serum potassium (LBXSKSI) | 4.01 | 0.35 | 4 | 3.8 | 4.2 | 3.2 | 7.3 |
| Lactate dehydrogenase (LBXSLDSI) | 136.5 | 34 | 132 | 115 | 152 | 82 | 1539 |
| LDH / HDL cholesterol ratio (LBXSLDSI_div_LBDHDD) | 2.8 | 1.1 | 2.6 | 2.1 | 3.3 | 0.7 | 40.5 |
| Serum sodium (LBXSNASI) | 139.3 | 2.38 | 139 | 138 | 141 | 133 | 161 |
| Serum osmolality (LBXSOSSI) | 278.7 | 5.38 | 278 | 275 | 282 | 266 | 323 |
| Serum phosphorus (LBXSPH) | 3.7 | 0.56 | 3.7 | 3.3 | 4.1 | 2.4 | 10.9 |
| Total bilirubin (LBXSTB) | 0.7 | 0.3 | 0.6 | 0.5 | 0.8 | 0.2 | 13.1 |
| Total protein (LBXSTP) | 7.2 | 0.49 | 7.2 | 6.9 | 7.5 | 6.1 | 11.3 |
| Triglycerides (LBXSTR) | 151.1 | 131.6 | 119 | 80 | 182 | 35 | 6057 |
| Uric acid (LBXSUA) | 5.42 | 1.46 | 5.3 | 4.4 | 6.3 | 2.6 | 18 |
| Uric acid / creatinine ratio (LBXSUA_div_LBXSCR) | 6.44 | 1.81 | 6.2 | 5.2 | 7.4 | 0.31 | 22 |
| Age (RIDAGEYR) | 49.6 | 18.1 | 49 | 34 | 64 | 20 | 85 |
| Age × BMI^2 (^RIDAGEYR BMXBMI) | 1444.7 | 619.1 | 1397.7 | 943.3 | 1881.1 | 358.4 | 5729.24 |
| Age × sex (RIDAGEYR RIAGENDR) | 75 | 37.7 | 66 | 46 | 98 | 20 | 170 |
| Age × BMI (RIDAGEYR_x_BMXBMI) | 1440.3 | 619.1 | 1392 | 940.5 | 1872 | 358.4 | 5729.2 |
| Age × sex (RIDAGEYR_x_RIAGENDR) | 75 | 37.7 | 66 | 46 | 98 | 20 | 170 |
| arthritis × average diastolic BP (arthritis_x_avg_di) | 18.2 | 31.5 | 0 | 0 | 46 | 0 | 101.3 |
| Average dietary index score (avg_di) | 67.9 | 18.5 | 70.7 | 62 | 78 | 0 | 101.3 |
| Mean of blood biomarker (blood_work_mean) | 63.8 | 18.1 | 61.5 | 53.7 | 70.5 | 3.2 | 643.7 |
| Median of blood biomarker (blood_work_median) | 54.7 | 21. | 52.5 | 41 | 65 | 3.2 | 269.5 |
| Standard deviation of blood biomarker (blood_work_std) | 64 | 20.9 | 61.3 | 53.6 | 70.6 | 2.1 | 1089.1 |
| Diabetes × BMI (diabetes_x_BMXBMI) | 3.9 | 10.8 | 0 | 0 | 0 | 0 | 84.4 |
| LDH used as a heart failure–related marker (hf_LBXSLDSI) | 4.9 | 27.9 | 0 | 0 | 0 | 0 | 779 |
| High cholesterol × BMI interaction (highchol_x_BMXBMI) | 9.5 | 14.4 | 0 | 0 | 25 | 0 | 84.4 |
| LDH level among participants with hypertension (hypertension_LBXSLDSI) | 49.7 | 71.4 | 0 | 0 | 122 | 0 | 1539 |
| Hypertension × BMI interaction (hypertension_x_BMXBMI)d | 10.8 | 15.4 | 0 | 0 | 26.6 | 0 | 130.2 |
| hypertension_x_RIDAGEYR | 21 | 30 | 0 | 0 | 52 | 0 | 85 |
| overweight_x_BMXBMI | 9.2 | 15.8 | 0 | 0 | 26 | 0 | 130.2 |
| overweight × average diastolic BP （overweight_x_avg_di） | 18.3 | 32 | 0 | 0 | 44.7 | 0 | 101.3 |

Table S1b. **Baseline characteristics—categorical variables** counts and percentages (n, % of total).

| **Variable** | **Total N = 51,698 (100%)** |
| --- | --- |
| AMEBICIDES | 83 (0.2%) |
| AMINOGLYCOSIDES | 10 (0.0%) |
| ANALGESICS | 6640 (12.8%) |
| ANOREXIANTS | 9 (0.0%) |
| ANTACIDS | 11 (0.0%) |
| ANTICOAGULANTS | 1124 (2.2%) |
| ANTICONVULSANTS | 2795 (5.4%) |
| ANTIDEPRESSANTS | 4800 (9.3%) |
| ANTIDIARRHEALS | 81 (0.2%) |
| ANTIDOTES | 10 (0.0%) |
| ANTIFUNGALS | 146 (0.3%) |
| ANTIHISTAMINES | 1146 (2.2%) |
| ANTIMETABOLITES | 198 (0.4%) |
| ANTIPSORIATICS | 5 (0.0%) |
| ANTIPSYCHOTICS | 686 (1.3%) |
| ANTIRHEUMATICS | 148 (0.3%) |
| ANTITUSSIVES | 72 (0.1%) |
| BRONCHODILATORS | 2262 (4.4%) |
| CALCIMIMETICS | 28 (0.1%) |
| CALCITONIN | 53 (0.1%) |
| CEPHALOSPORINS | 239 (0.5%) |
| DECONGESTANTS | 14 (0.0%) |
| DIURETICS | 4543 (8.8%) |
| ETHNICITY_1 | 8820 (17.1%) |
| ETHNICITY_2 | 4306 (8.3%) |
| ETHNICITY_3 | 22927 (44.3%) |
| ETHNICITY_4 | 11135 (21.5%) |
| ETHNICITY_5 | 4510 (8.7%) |
| EXPECTORANTS | 48 (0.1%) |
| IMMUNOSTIMULANTS | 35 (0.1%) |
| LAXATIVES | 271 (0.5%) |
| LEPROSTATICS | 9 (0.0%) |
| PENICILLINS | 623 (1.2%) |
| PSORALENS | 2 (0.0%) |
| QUINOLONES | 235 (0.5%) |
| RIAGENDR | 26756 (51.8%) |
| SMQ020 | 23544 (45.5%) |
| SULFONAMIDES | 197 (0.4%) |
| TETRACYCLINES | 108 (0.2%) |
| VASODILATORS | 256 (0.5%) |
| VASOPRESSORS | 8 (0.0%) |
| VITAMINS | 206 (0.4%) |
| Angina | 1476 (2.9%) |
| Liver disease | 1973 (3.8%) |
| Arthritis | 14001 (27.1%) |
| Asthma | 7044 (13.6%) |
| Overall cancer | 4875 (9.4%) |
| Cancer bladder | 132 (0.3%) |
| Cancer blood | 15 (0.0%) |
| Cancer bone | 42 (0.1%) |
| Cancer brain | 24 (0.0%) |
| Cancer breast | 747 (1.4%) |
| Cancer cervix | 335 (0.6%) |
| Cancer colon | 355 (0.7%) |
| Cancer colorectal | 377 (0.7%) |
| Cancer endometrial | 569 (1.1%) |
| Cancer esophageal | 30 (0.1%) |
| Cancer gallbladder | 1 (0.0%) |
| Cancer kidney | 97 (0.2%) |
| Cancer larynx | 27 (0.1%) |
| Cancer leukemia | 58 (0.1%) |
| Cancer liver | 35 (0.1%) |
| Cancer lung | 140 (0.3%) |
| Cancer lymphoma | 116 (0.2%) |
| Cancer melanoma | 331 (0.6%) |
| Cancer mouth/tongue/kip | 28 (0.1%) |
| Cancer nervous | 1 (0.0%) |
| Cancer ovary | 136 (0.3%) |
| Cancer pancreas | 12 (0.0%) |
| Cancer prostate | 789 (1.5%) |
| Cancer rectum | 30 (0.1%) |
| Cancer skin general | 427 (0.8%) |
| Cancer skin nonmelanoma | 797 (1.5%) |
| Cancer soft tissue | 11 (0.0%) |
| Cancer stomach | 38 (0.1%) |
| Cancer testis | 32 (0.1%) |
| Cancer thyroid | 111 (0.2%) |
| Cancer urologic | 987 (1.9%) |
| Cancer uterus | 242 (0.5%) |
| Coronary Heart Disease | 2170 (4.2%) |
| Chronic burden (no condition) | 30261 (58.5%) |
| Chronic burden (one) | 15780 (30.5%) |
| Chronic burden (two) | 4910 (9.5%) |
| Chronic burden (three or more) | 747 (1.4%) |
| Diabetes | 6337 (12.3%) |
| Heart Attack | 2258 (4.4%) |
| Heart Failure | 1714 (3.3%) |
| High Cholesterol | 16316 (31.6%) |

Table S2. Demographic between High-fat diet vs. Non high-fat diet group (cut-off 20).

| Characteristic | Non high-fat diet (n=8,129) | High-fat diet  (n=33,238) |
| --- | --- | --- |
| Age (Mean ± SD) | 50.5 ± 18.3 | 49.4 ± 18.1 |
| BMI (Mean ± SD) | 28.4 ± 6.2 | 29.3 ± 7.0 |
| Age group |  |  |
| 18-39 | 2653 (32.6%) | 11483 (34.5%) |
| 40-59 | 2538 (31.2%) | 10653 (32.1%) |
| >60 | 2929 (36.0%) | 11102 (33.4%) |
| Sex |  |  |
| Female | 4256 (52.4%) | 17183 (51.7%) |
| Male | 3864 (47.5%) | 16055 (48.3%) |
| Race/Ethnicity |  |  |
| Mexican American | 1770 (21.8%) | 5360 (16.1%) |
| Non-Hispanic Black | 1447 (17.8%) | 7444 (22.4%) |
| Non-Hispanic White | 2953 (36.3%) | 15327 (46.1%) |
| Other Hispanic | 929 (11.4%) | 2512 (7.6%) |
| Other Race | 1021 (12.6%) | 2595 (7.8%) |
| Comorbidities |  |  |
| Overweight | 1813 (22.3%) | 9168 (27.6%) |
| Hypertension | 2746 (33.8%) | 11735 (35.3%) |
| Cancer | 684 (8.4%) | 3174 (9.5%) |

Table S3. Temporal validation performance of Random Forest model across three high-fat diet cut-off thresholds.

|  | F1-Score | Recall | Precision |
| --- | --- | --- | --- |
| cutoff 10 | 0.379 ± 0.007 | 0.489 ± 0.013 | 0.309 ± 0.006 |
| cutoff 15 | 0.604 ± 0.006 | 0.644 ± 0.01 | 0.569 ± 0.004 |
| cutoff 20 | 0.732 ± 0.009 | 0.653 ± 0.014 | 0.834 ± 0.003 |

Tabel S4. Post-hoc power analysis.

|  | Odds Ratio | p-value | Statistical Power |
| --- | --- | --- | --- |
| Cancer | 1.23 [1.03, 1.47] | 0.02 | 0.999 |
| Colorectal cancer | 1.17 [0.68, 2.00] | 0.57 | 0.233 |
| Urologic cancer | 1.18 [0.81, 1.73] | 0.39 | 0.501 |
| Breast cancer | 0.86 [0.59, 1.27] | 0.46 | 0.388 |
| Lung cancer | 1.21 [0.41, 3.60] | 0.73 | 0.142 |
| Prostate cancer | 1.13 [0.74, 1.73] | 0.58 | 0.509 |

Method S1. Practical deployment considerations.

Although our modeling approach relies on standard supervised learning methods rather than novel algorithms, our results suggest a practical pathway for deploying a scalable computable diet phenotype in routine data systems. In an EHR setting, predicted diet phenotype scores could be generated automatically at the patient or encounter level using routinely collected variables and integrated into (i) population health registries for risk stratification, (ii) clinical decision support to prompt dietary assessment or referral, or (iii) research data marts to enable downstream studies of diet-related outcomes at scale. Prior to clinical use, several implementation steps are essential: external validation in independent health systems, calibration assessment and recalibration as needed, evaluation of performance across demographic subgroups, and monitoring for dataset shift over time. Given the nontrivial missingness and evolving practice patterns in real-world data, we recommend a lightweight model monitoring strategy (periodic performance checks, drift detection, and threshold re-tuning) and transparent model documentation to support reproducibility and safe use.
